# Poisoning by Strychnine

**Published:** 1867-09

**Authors:** Stacy Hemenway

**Affiliations:** Eugene City, Oregon


ARTICLE XLI.

### POISONING BY STRYCHNINE.—RECOVERY.

By STACY HEMENWAY, Eugene City, Oregon.

Having noticed the recovery of two cases of poisoning from strychnine, recently reported through your valuable journal, one by S. A. McWilliams, A.M., M.D., and the other by Julien S. Sherman, M.D., both of Chicago, Ill., I deem it of equal importance to relate another case of recovery from poisoning by strychnine, which occurred seven miles south of this place, on the 5th of June, 1867.

James G., a resident of Lane County, Oregon, *æt.* 28, swallowed a quahtity of strychnine, accidentally, at about half-past 8 o'clock in the morning. It occurred about as follows: The patient had been in the habit of putting strychnine out to kill the "varmints," as he termed them, and had carelessly left upon the table a moistened teaspoon which he had been using in the finely powdered substance, a considerable quantity adhering to the spoon.

On the morning in question he had taken up a pail to go out and milk, but discovered before he started, (he perhaps being somewhat under the influence of an alcoholic stimulus at the time,) a cup on the table containing milk, which he desired to drink. He picked up this spoon with which to stir the cream into the milk, and thus thoughtlessly swallowed the mixture. He stated that immediately after taking the poison everything seemed to turn green, and that he suddenly fell to the floor, with inability to move his limbs, while his whole muscular system was in a state of tremor. He remained unable to move until about 2 o'clock P.M., and was unfortunately entirely alone. The nearest house was perhaps a fourth of a mile off, which he succeeded in reaching in the course of two hours by crawling and walking at intervals between the spasms, which were of frequent occurrence. Vomiting occurred once on the way. A messenger was now sent in haste for me. I reached the patient at about 8 o'clock P.M., nearly twelve hours after the swallowing of the poison, and found him suffering from the characteristic symptoms of poisoning by strychnine, having spasms, extensive, frequent, and severe, and excessive spitting of frothy saliva. About five minutes after my arrival a severe convulsive paroxysm occurred, which lasted twenty minutes. These violent spasms occurred at intervals of about half an hour, and about four thrills per minute passing through the system. He was suffering, also, from spasmodic contraction of the muscles of the chest about four times in a minute, with a feeling of impending suffocation. There was marked rigidity of the cervical muscles, the jaws set, and he complained of a sense of constriction of the fauces, with difficulty of swallowing. The extremities were cool; countenance anxious; pulse 110 to the minute, and the mind clear and sensible. Having with me the cannabis indica, in the form of the alcoholic extract, (the tincture being unobtainable at the time.) I immediately administered from 4 to 5 grains in pilular form, and repeated the dose in five minutes; then four similar doses at intervals of ten minutes; afterwards three such doses at intervals of fifteen minutes, with a rapid improvement of the symptoms. Symptoms of the intoxicating effects of this drug began now to be slightly manifested. The administration of the remedy was suspended for an hour, and then resumed again, in gradually diminished quantities, alternated with spts. camphor in drachm doses, every fifteen minutes, until four doses of each had been administered, when the patient began to be comparatively quiet, with a strong tendency to sleep. He began to sleep well at 2 o'clock A.M.; left directions with an attendant to administer a similar dose of each, one hour apart. During the administration of these remedies, the spasms gradually grew less violent, together with a gradual diminution, in severity, of the tremulous movements running through the system. The patient slept well, until late in the morning, when he awoke, complaining of extreme exhaustion, with general muscular soreness. A very slight thrilling sensation running through the system still prevailed. The patient was directed to take one 3 grain pill of the ext. cannabis indica every two hours for 6 hours, alternated with drachm doses of the spts. camphor. Nutritious diet with tonics soon rendered recovery complete.

